# Pharmacists’ knowledge, attitude and involvement in palliative care in selected tertiary hospitals in southwestern Nigeria

**DOI:** 10.1186/s12904-019-0492-8

**Published:** 2019-11-29

**Authors:** Rasaq ADISA, Aderonke Tolulope ANIFOWOSE

**Affiliations:** 1 Department of Clinical Pharmacy and Pharmacy Administration, Faculty of Pharmacy, University of Ibadan, Ibadan, Nigeria; 2 University Health Services, University of Ibadan, Ibadan, Nigeria

**Keywords:** Hospital pharmacists, Knowledge, attitude and involvement, Palliative care, Nigeria

## Abstract

**Background:**

The growing number of people living with life-limiting illness is a global health concern. This study therefore aimed to explore the involvement of pharmacists in selected tertiary hospitals in Nigeria in palliative care (PC). It also sought to evaluate their knowledge and attitude to PC as well as factors that hinder pharmacists’ participation in PC.

**Method:**

Questionnaire-guided survey among pharmacists working in three-tertiary hospitals in southwestern Nigeria. The self-administered questionnaire comprised 18-item general knowledge questions related to PC, attitude statements with 5-point Likert-scale options and question-items that clarify extent of involvement in PC and barriers to participation. Overall score by pharmacists in the knowledge and attitude domains developed for the purpose of this study was assigned into binary categories of “adequate” and “inadequate” knowledge (score > 75% versus≤75%), as well as “positive” and “negative” attitude (ranked score > 75% versus≤75%), respectively. Descriptive statistics, Mann-Whitney-U and Kruskal-Wallis tests were used for analysis at *p* < 0.05.

**Results:**

All the 110 pharmacists enrolled responded to the questionnaire, given a response rate of 100%**.** Overall, our study showed that 23(21.1%) had adequate general knowledge in PC, while 14(12.8%) demonstrated positive attitude, with 45(41.3%) who enjoyed working in PC. Counselling on therapy adherence (100;90.9%) was the most frequently engaged activity by pharmacists; attending clinical meetings to advise health team members (45;40.9%) and giving educational sessions (47;42.7%) were largely cited as occasionally performed duties, while patient home visit was mostly cited (60;54.5%) as a duty not done at all. Pharmacists’ unawareness of their need in PC (86;79.6%) was a major factor hindering participation, while pharmacists with PC training significantly felt more relaxed around people receiving PC compared to those without training (*p* = 0.003).

**Conclusion:**

Hospital pharmacists in selected tertiary care institutions demonstrate inadequate knowledge, as well as negative attitude towards PC. Also, extent of involvement in core PC service is generally low, with pharmacists’ unawareness of their need in PC constituting a major barrier. Thus, a need for inclusion of PC concept into pharmacy education curriculum, while mandatory professional development programme for pharmacists should also incorporate aspects detailing fundamental principles of PC, in order to bridge the knowledge and practice gaps.

## Background

The growing number of people living with life-limiting illness is a global health concern that may necessitate a paradigm shift towards provision of palliative care service by all healthcare professionals regardless of the practice settings [1–4]. Current estimate suggests that approximately 75% of people approaching the end-of-life may benefit from palliative care [4], with international and local evidence demonstrating the impact of palliative care on patient outcomes, caregiver and healthcare [2]. Palliative care is a patient- and family-centred approach to care focusing on quality of life and relief of symptoms [5–7], thus, it is a complex health discipline that requires extensive collaboration and teamwork among healthcare professionals, patients and caregivers [6, 8, 9]. Studies have identified the palliative care population as one of the groups who are at the highest risk of medication misadventure and adverse events, and consequently increased hospital admissions [10, 11]. Adam et al [12] reported that almost one-third of patients with cancer access out-of-hours primary medical care due to poorly controlled pain. In essence, patients requiring palliative care typically have complicated and high risk medication regimen such as opioids that require frequent adjustment and monitoring [13, 14], thereby making the pharmacist a highly desirable member of palliative care team whose contribution can potentially improve the patient medication management and reduce the risk of non-adherence [15–17]. Published reports especially from developed countries have identified different roles played by pharmacists in palliative care with benefits and effectiveness of service offered in this context [15–23]. However, in most developing countries, palliative care is still an emerging medical specialty that require healthcare providers’ contribution and participation [24–27]. In Nigeria for instance, palliative care is in its early stage of development [9, 24, 25, 27], with services mainly limited to patients who attend the tertiary hospitals [24], while pharmacists were recognized among the least considered member of the healthcare team providing palliative care service [27–30]. In addition, the Benchmark for Minimum Academic Standard from the two major regulatory agencies for pharmacy education in Nigeria, that is the National Universities Commission and the Pharmacists Council of Nigeria does not include aspects on palliative care in the curriculum contents. This is in spite of the pharmacists’ essential role in medication therapy management especially for patients with complex chronic regimen [15–23]. Nevertheless, studies from developed countries have generally identified reasons for low involvement of pharmacists in palliative care to include inadequate knowledge and skills, deficient education and training in palliative care [16, 30–32], as well as attitude and belief towards palliative care [16, 30, 33]. In Nigeria however, there is dearth of evidence-based research that directly examine the extent of pharmacists’ involvement in palliative care as well as barriers to participation. This study therefore aimed to comprehensively evaluate knowledge, attitude and involvement of hospital pharmacists in selected tertiary healthcare institutions in southwestern Nigeria in palliative care, while factors that may hinder their involvement in palliative care were also explored. The information obtained from this study may help in identifying areas of focus for future advocacy and intervention to address the practice gaps.

## Method

### Study design

This study was a questionnaire-guided survey among pharmacists working in three selected tertiary hospitals with established palliative care services between February and April, 2017.

### Study setting

Pharmacy departments of selected hospitals namely University College Hospital (UCH) Ibadan, Obafemi Awolowo University Teaching Hospitals Complex (OAUTH) Ile-Ife and Federal Medical Centre (FMC) Abeokuta. The UCH is a 900-bed premier teaching hospital in Nigeria, affiliated with University of Ibadan; OAUTHC is a 600-bed teaching hospital affiliated with Obafemi Awolowo University, while FMC is a 350-bed tertiary care hospital established with a special focus on chronic disease management. The hospitals are federal healthcare institutions strategically located within the southwest geopolitical zone in the country, and are major sites for undergraduate and post-graduate residency training for physicians, as well as clinical training for other categories of healthcare practitioners including pharmacists, nurses and other ancillary healthcare personnel.

### Study population

Post-intern pharmacists working in the three selected tertiary hospitals.

### Inclusion and exclusion criteria

Post-intern hospital pharmacists who consented to participate in the study were enrolled, while those with less than 1 year experience in hospital practice were excluded.

### Sample size determination

Number of eligible pharmacists from each hospital was obtained from the respective institution as: UCH = 70, OAUTHC = 38 and FMC = 12, given a total estimated population of 120 pharmacists in the three selected facilities. Based on the estimated population at 95% confidence level and 5% margin of error, a sample size of 92 was obtained using Yamane sample size formula [34]. Adjusting for a 10% non-response rate gave a target sample population of approximately 102. The proportion of pharmacists recruited from each facility was subsequently guided as follows: $\mathrm{UCH}\;\left(\frac{70}{120}X\;102=60\right)$, $\mathrm{OAUTHC}\;\left(\frac{38}{120}\;X\;102=32\right)$ and $\mathrm{FMC}\;\left(\frac{12}{120}\;X\;102=10\right)$, where the numerator denotes number of eligible pharmacists in the respective hospital, while the denominator represents the estimated population. The total of 110 copies of questionnaire were given to pharmacists in the three selected hospitals**.**

### Data collection instrument

The main instrument used for data collection was a semi-structured questionnaire developed by the investigators following extensive review of relevant studies [6, 7, 24, 30], as well as utilizing previous experience from palliative care training. The questionnaire consisted of five sections. Section A captured demographic characteristics, years of experience in hospital practice, previous training in palliative care, as well as cadre/rank. Section B contained 18-item questions that evaluated general knowledge in palliative care. Section C evaluated opinion on relevant attitude-related statements toward palliative care which was adapted from Pitzen (2009) [35]. The attitude questions had a 5-point Likert scale response option ranging from strongly disagree (1) to strongly agree (5) for positive statements (1–6), and a reversed score for the negative statements (7–13). Section D contained item-statements that clarified extent of involvement in some palliative care services in their respective practice site, while Section E contained questions that explored possible factors that may hinder involvement in palliative care (see additional file 1).

### Pretest and content validation

The questionnaire was assessed for content validity by two pharmacists in academia chosen from the department of Clinical Pharmacy and Pharmacy Administration, University of Ibadan, to ascertain the comprehensiveness of question-items *vis-à-vis* the study objectives, as well as ensuring that there are no ambiguous questions or statements. Subsequently, the questionnaire was given to three pharmacists chosen from a state-owned specialist hospital notable for chronic disease management, to ascertain the ease of comprehension of the item-statements. Feedback from the pretest and content validation led to few modifications in the questionnaire such as rephrasing of a dichotomous Yes/No response option as a “true” or “false” answer for questions on general knowledge in palliative care. Also, the re-designing of attitude statements from a Yes/No answer into a Likert scale response option to ensure clarification of opinion.

### Sampling and data collection procedure

Eligible hospital pharmacists were enrolled using purposive sampling approach by visiting individual pharmacist in their respective practice site. Objectives of the study were explained to every pharmacist after which voluntary verbal informed consent was obtained to signify intention to participate in the study. The questionnaire was self-administered by all consented pharmacists and retrieved within 25–30 min of completion of the questionnaire. Anonymity and confidentiality of response were assured, while participation was entirely voluntary.

### Data analysis

Data obtained were sorted, coded and entered into Microsoft Excel spreadsheet file for ease of data management, and subsequently the computed data were exported into SPSS version 21.0 for analysis. Descriptive statistics including frequency and percentage were used to summarise the data. In this study, the overall score by pharmacists in the knowledge and attitude domains developed for the purpose of this study was converted into percentage to ensure uniformity in the scores. In the knowledge domain, a total score > 75% was considered as “adequate” knowledge, while score ≤ 75% signified “inadequate” knowledge. In other word, a percent score > 75% indicates a raw score of > 13 out of the 18 questions that evaluated the general knowledge in palliative care; > 6 out of 8 questions on knowledge of diseases that mostly require palliative care service; and > 5 out of 7 questions on knowledge of palliative care team composition. In the attitude domain, a total ranked score > 75% was considered as “positive” attitude, that is, a raw score of ≥49 out of maximum obtainable score of 65, while a ranked score ≤ 75% signified “negative” attitude towards palliative care. The cut-off criteria for the binary categorization was adapted from Bloom’s cut-off point criteria, as well as review of other related studies [36, 37]. Pearson Chi-square (X^2^) test was used to investigate association between demographic characteristics, as well as years of experience in hospital practice and general knowledge in palliative care. Mann-Whitney U (MWU) test was used to evaluate association among pharmacists with or without palliative care training and opinion on attitude-related statements, while relationship between hospital of practice by pharmacists and attitude towards palliative care was investigated using Kruskal-Wallis (K-W) test at *p* < 0.05 level of statistical significance.

## Results

**Fig. 1:**
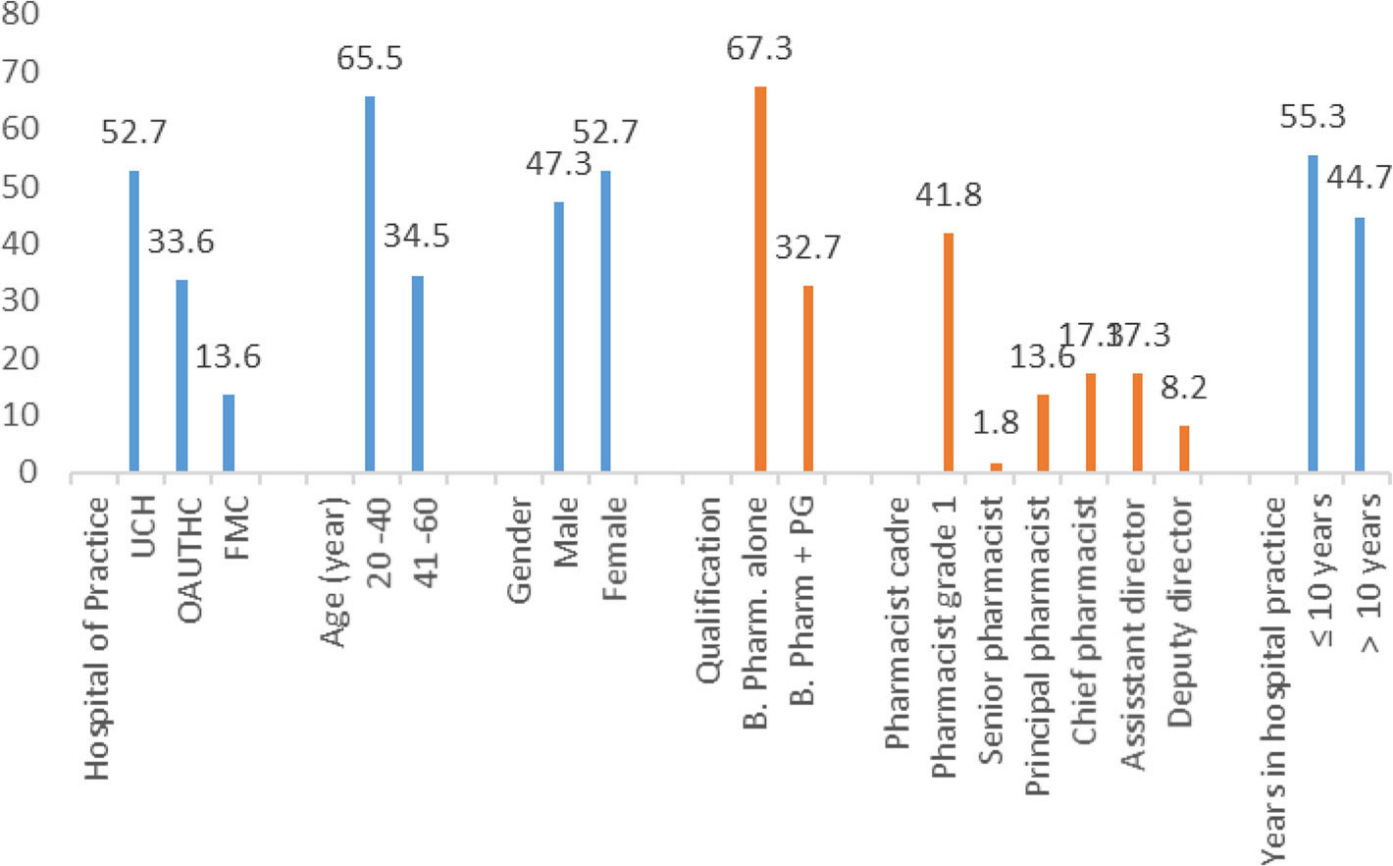
Demographic characteristics and years of experience in hospital practice among pharmacists (Demographic variables versus percent)

All the 110 pharmacists who were enrolled responded to the questionnaire, given a response rate of 100%. Fifty-eight (52.7%) pharmacists participated from UCH, 37 (33.6%) from OAUTHC and 15 (13.6%) from FMC. Details of demographic characteristics and years of experience in hospital practice are shown in Fig. 1. The majority, 72 (65.5%) were in the age range of 20–40 years, 58 (52.7%) were females and 35 (32.7%) had additional postgraduate qualification. Participants (46; 41.8%) were mostly in the pharmacist grade one cadre (i.e. within 1 to 3 years of full-time hospital employment). Seventy-seven (58.8%) of the pharmacists had worked in the general outpatient pharmacy unit within the last 2 years prior to the commencement of this study, 16 (12.2%) in ward pharmacy unit, 10 (7.6%) in medical outpatient, 8 (6.1%) in oncology, 6 (4.6%) in paediatric unit, 5 (3.8%) in virology, 3 (2.3%) in surgical outpatient, 2 (1.5%) in haematology and immunology, 2 (1.5%) in radiopharmacy/nuclear pharmacy, while one (0.8%) each had worked in psychiatry and as a member of palliative care team. Eighty-three (75.7%) had encountered patients requiring palliative care, while 21 (19.1%) had previously attended a palliative care related training.

Table 1 shows the general knowledge of pharmacists in palliative care. Ninety-one (85.0%) understood that long-time use of opioids for palliative care patients does not often results in addiction. One hundred and one (93.5%) felt that the goals of palliative care are consistent with the philosophy of pharmaceutical care, while 67 (64.2%) had a misconception that medication therapy is the cornerstone of all symptom control in palliative care. Overall, 23 (21.1%) had score > 75% indicating “adequate” knowledge of palliative care and its principles (see Table 1). Table 2 provides information on the knowledge of pharmacists in relation to palliative care team composition, as well as diseases that mostly require palliative care service. Sixty-five (59.1%) were absolutely correct in citing all the listed categories of professionals as a possible member of palliative care team (see Table 2).

**Table 1:** Response to the 18-items general knowledge questions about palliative care and its principles among pharmacists

Statement	True, n (%)	False, n (%)
1.Palliative care involves provision of care only to patients who have no curative treatment available (*n* = 108)	43 (39.8)	65 (60.2)^a^
2. Non-medical practitioners are active participant in palliative care (*n* = 108)	63 (58.3)^a^	45 (41.7)
3. Palliative care is to be provided by doctors and nurses alone (*n* = 109)	3 (2.8)	106 (97.2)^a^
4. Palliative care is required only for patients who are near death (*n* = 108)	18 (16.7)	90 (83.3)^a^
5. Palliative care only involves pain management (*n* = 108)	25 (23.1)	83 (76.9)^a^
6. Palliative care involves providing patients with relief from their symptoms (*n* = 109)	66 (60.6)^a^	43 (39.4)
7. Regular opioids intake should not be combined with non-steroidal anti-inflammatory drugs for palliative care patients (*n* = 99)	45 (45.5)	54 (54.5)^a^
8. Long term use of opioids for palliative care patients does not often induce addiction (*n* = 107)	91 (85.0)^a^	16 (15.0)
9. Palliative care should not be provided alongside anti-retroviral treatment (*n* = 99)	5 (5.1)	94 (94.9)^a^
10. One of the goals of pain management in palliative care is to get good night sleep (*n* = 108)	14 (13.0)^a^	94 (87.0)
11. Benzodiazepines should be effective for controlling delirium in palliative care patients (*n* = 93)	71 (76.3)^a^	22 (23.7)
12. Palliative care does not involve maintaining patient medication profile overtime (*n* = 105)	8 (7.6)	97 (92.4)^a^
13. Palliative care should not be provided in conjunction with curative care at the time of diagnosis of a potential life-limiting illness (*n* = 101)	14 (13.9)	87 (86.1)^a^
14. The goals of palliative care and pharmaceutical care are consistent (*n* = 108)	101 (93.5)^a^	7 (6.5)
15. Medication therapy is the cornerstone of all symptom control in palliative care (*n* = 104)	67 (64.4)	37 (35.6)*
16. Involvement in palliative care activities by pharmacists may decrease the need for medical emergencies (*n* = 103)	103 (100.0)^a^	0 (0.0)
17. Pharmacist in palliative care should be less concerned about monitoring non-prescription medication use for safety and effectiveness (*n* = 107)	3 (2.8)	104 (97.2)^a^
18. Pharmacists in palliative care communicate with pharmaceutical manufacturers to determine the availability of nonstandard dosage forms (*n* = 99)	61 (61.6)^a^	38 (38.4)
Cut-off for overall percent score	n (%)	Remark
> 75	23 (21.1)	Adequate
≤75	86 (78.9)	Inadequate

^a^ = Correct answer. Maximum obtainable score = 18; % individual score = score obtained by an individual ÷ total obtainable score × 100, n = number

**Table 2:** Pharmacists’ knowledge of diseases requiring palliative care and palliative care team composition

Variables (*n* = 110)	Response category	
Disease requiring palliative care service	Yes, n (%)	No, n (%)
Cardiovascular diseases	60 (54.5)^a^	50 (45.5)
HIV/AIDS	95 (86.4)^a^	15 (13.6)
Renal diseases	83 (75.5)^a^	27 (24.5)
Peptic ulcer diseases	24 (21.8)	86 (78.2)^a^
Asthma	32 (29.1)	78 (70.9)^a^
End stage pulmonary diseases	90 (81.8)^a^	20 (18.2)
Parkinson disease	62 (56.4)^a^	48 (43.6)
Dementia	62 (56.4)^a^	48 (43.6)
Cut-off for overall percent score	Frequency (%)	Remark
> 75	21 (19.1)	Adequate knowledge
≤ 75	89 (80.9)	Inadequate knowledge
Palliative care team composition includes:	Yes, n (%)	No, n (%)
Physician	107 (97.3)	3 (2.7)
Pharmacist	107 (97.3)	3 (2.7)
Nurse	105 (95.5)	5 (4.5)
Psychologist	94 (85.5)	16 (14.5)
Chaplain	75 (68.2)	35 (31.8)
Social worker	90 (81.8)	20 (18.2)
All of the above	65 (59.1)^b^	45 (40.9)
Cut-off for overall percent score	Frequency (%)	Remark
> 75	73 (66.4)	Adequate knowledge
≤ 75	37 (33.6)	Inadequate knowledge

^a^Correct answer, ^b^ = most correct answer, n = number, maximum obtainable score for questions on knowledge of diseases requiring palliative care = 8, and palliative care team composition = 7; % individual score = score obtained by an individual ÷ total obtainable score × 100. Cancer is excluded as a response option because it is well-known that patients with cancer will require palliative care

Response of pharmacists to attitude-related statements is shown in Table 3. Forty-five (41.3%) enjoyed working in palliative care, 61 (57.0%) felt confident in managing symptoms in palliative care, while 90 (83.3%) believed that it is rewarding to work with people receiving palliative care. A total of 14 (12.8%) demonstrated “positive” attitude towards palliative care (see Table 3). Table 4 shows the perceived extent of pharmacists’ involvement in general patient and palliative care services. Counselling on therapy adherence (100; 90.9%), as well as ensuring complete labelling and direction for medication usage (100; 90.9%) were the most frequently engaged activities. Giving educational sessions (47; 42.7%) and attending clinical meetings to advise other health team members (45; 40.9%) were largely cited as occasionally performed duties, while patient home visit was mostly cited (60; 54.5%) as a duty not done at all (see Table 4). Perceived factors limiting involvement in palliative care are shown in Table 5. Pharmacists’ unawareness of their need in palliative care (86; 79.6%) topped the list of factors hindering participation, while non-accessibility to patients’ medication profile (72; 70.6%), inadequate knowledge of palliative care among pharmacists (65; 60.2%), as well as fear of being around people with terminal illness (32; 29.9%) were also cited as barriers (see Table 5).

**Table 3:** Assessment of pharmacists’ attitude towards palliative care

Attitude statement related to palliative care	SD (1) n (%)	D (2) n (%)	U (3) n (%)	A (4) n (%)	SA (5) n (%)
1.I enjoy working in palliative care (*n* = 109)	16 (14.7)	40 (36.7)	8 (7.3)	32 (29.4)	13 (11.9)
2. I feel relaxed around people receiving palliative care (*n* = 105)	5 (4.8)	9 (8.6)	48 (45.7)	36 (34.3)	7 (6.7)
3. I feel confident in managing symptoms in palliative care (*n* = 106)	1 (0.9)	9 (8.5)	35 (33.0)	52 (49.1)	9 (8.5)
4. I feel comfortable talking about dying to a patient receiving palliative care (*n* = 105)	19 (18.1)	39 (37.1)	34 (32.4)	10 (9.5)	3 (2.9)
5. I don’t mind working in palliative care despite its involvement in managing people with life-limiting illness (*n* = 108)	4 (3.7)	12 (11.1)	58 (53.7)	25 (23.1)	9 (8.3)
6. There is a difference between providing palliative care service and normal hospital care (*n* = 108)	35 (32.4)	54 (50.0)	10 (9.3)	7 (6.5)	2 (1.9)
	SD (5)	D (4)	U (3)	A (2)	SA (1)
7. I am not comfortable touching people with terminal illness (*n* = 107)	31 (29.0)	51 (47.7)	18 (16.8)	6 (5.6)	1 (0.9)
8. I don’t believe that pharmacists have any role to play as a member of palliative care team (*n* = 108)	68 (62.9)	29 (26.9)	8 (7.4)	1 (0.9)	2 (1.9)
9. I feel frustrated because I do not know how to help people receiving palliative care (*n* = 107)	21 (19.6)	53 (49.5)	25 (23.4)	8 (7.5)	0 (0.0)
10. It is not rewarding to work with people who are receiving palliative care (*n* = 108)	39 (36.1)	51 (47.2)	11 (10.2)	4 (3.7)	3 (2.8)
11. I am not familiar with pain symptoms necessary for palliative care (*n* = 105)	7 (6.7)	43 (41.0)	18 (17.1)	33 (31.4)	4 (3.8)
12. Working with terminally ill patients is sad and depressing (*n* = 107)	7 (6.5)	26 (24.3)	22 (20.6)	41 (38.3)	11 (10.3)
13. Emotionally I don’t fit into palliative care (*n* = 103)	18 (17.5)	42 (40.8)	32 (31.1)	7 (6.8)	4 (3.8)
Cut-off for overall percent attitude score	Frequency (%)		Remark		
> 75	14 (12.8)		Positive attitude		
≤ 75	95 (87.2)		Negative attitude		

Maximum obtainable score = 65; % individual score = score obtained by an individual ÷ total obtainable score × 100. Statements 1 to 6 are positive attitude items, and 7 to 13 are negative attitude items. Strongly disagree (SD), Disagree (D), Undecided (U), Agree (A), Strongly agree (SA), n = number

**Table 4:** Pharmacists’ perceived extent of involvement in general patient care and palliative care services

My involvement in patient care as a pharmacist entails:	Not at all n (%)	Rarely n (%)	Occasionally n (%)	Frequently n (%)
1.Explain misconceptions about addictive medication (*n* = 110)	7 (6.4)	18 (16.4)	37 (33.6)	48 (43.6)
2. Visit patients’ homes to communicate directly with patients and their caregivers and to make necessary assessments (*n* = 110)	60 (54.5)	36 (32.7)	11 (10.0)	3 (2.7)
3. Monitor patients’ medication profile for safety and effectiveness (*n* = 109)	2 (1.8)	31 (28.4)	33 (30.3)	43 (39.4)
4. Provide patients with essential medications that ensure continuous symptom control (*n* = 110)	14 (12.7)	6 (5.5)	15 (13.6)	75 (68.2)
5. Attend clinical meetings to advise other members of healthcare team about medication therapy (*n* = 110)	12 (10.9)	22 (20.0)	45 (40.9)	31 (28.2)
6. Advise clinical team on dosage forms and adjustments, routes of administration, costs, and availability of various drug products (*n* = 110)	15 (13.6)	13 (11.8)	42 (38.2)	40 (36.4)
7. Give educational sessions (*n* = 110)	15 (13.6)	23 (20.9)	47 (42.7)	25 (22.7)
8. Advise members of the clinical team about the potential for toxicity and interactions with dietary supplements and alternative therapies (*n* = 110)	13 (11.8)	34 (30.9)	37 (33.6)	26 (23.6)
9. Ensure that all medication labeling is complete and understandable by patients and their caregivers (*n* = 110)	4 (3.6)	4 (3.6)	2 (1.8)	100 (90.9)
10. Communicate with patients about the importance of adhering to the prescribed drug regimen (*n* = 110)	0 (0.0)	4 (3.6)	6 (5.5)	100 (90.9)
11. Monitor all prescription and nonprescription medication use (*n* = 108)	3 (2.8)	7 (6.5)	38 (35.2)	60 (55.5)
12. Counsel patients about potential toxicity of alternative and complementary therapies (*n* = 109)	2 (1.8)	9 (8.3)	35 (32.1)	63 (57.8)
13. Extemporaneous preparation of non-conventional dosage forms for ease of administration to patients (*n* = 109)	4 (3.7)	5 (4.6)	28 (25.7)	72 (66.1)
14. Prepare flavouring medications to promote compliance (*n* = 103)	5 (4.9)	16 (15.5)	40 (38.8)	42 (40.8)
15. Address issues on cost of patients’ medications (*n* = 110)	6 (5.5)	12 (10.9)	43 (39.1)	49 (44.5)
16. Ensure that drug disposal is in compliance with federal and state drug control and environmental protection laws and regulations (*n* = 103)	3 (2.9)	16 (15.5)	26 (25.2)	58 (56.3)

*n* Number

**Table 5:** Perceived factors limiting pharmacists’ involvement in palliative care

General factors	Yes, n (%)	No, n (%)	Don’t know, n (%)
Lack of awareness of the need for pharmacists in palliative care (*n* = 108)	86 (79.6)	17 (15.7)	5 (4.6)
Lack of access to patients’ medication profile (*n* = 102)	72 (70.6)	21 (20.6)	9 (8.8)
Inadequate knowledge of palliative care among pharmacists (*n* = 108)	65 (60.2)	29 (26.9)	14 (13.0)
Confusion of role in palliative care (*n* = 106)	62 (58.5)	30 (28.3)	14 (13.2)
Inadequate knowledge of concept of palliative care (*n* = 107)	60 (56.1)	37 (34.6)	10 (9.3)
Lack of reimbursement (*n* = 109)	48 (44.0)	39 (35.8)	22 (20.2)
Lack of pharmacists’ interest to work in palliative care (*n* = 107)	45 (42.1)	45 (42.1)	17 (15.9)
Fear of being around people with terminal illness (*n* = 107)	32 (29.9)	49 (45.8)	26 (24.3)
Belief that there could be a spiritual backlash from engaging in palliative care (*n* = 105)	15 (14.3)	67 (63.8)	23 (21.9)
Other suggested factors	Frequency	Percent	
Rivalry among healthcare workers	4	3.6	
Non-inclusion of palliative care in pharmacists’ routine rotations	2	1.8	
Inadequate professional work environment	1	0.9	

*n* Number

Table 6 shows relationship among pharmacists with or without palliative care training and opinion on attitude-related statements. Pharmacists who had training in palliative care were mostly found to be familiar with pain symptoms in palliative care (Mean rank [MR] = 69.24) compared to MR of 47.59 among those without training [MWU = 499.000, two-tailed *p* value = 0.002] (see Table 6).

**Table 6:** Association among pharmacists with or without palliative care training and attitude-related statements toward palliative care

	Previous training	N	Mean Rank	MW Up-value
1.I enjoy working in palliative care	Yes	21	30.79	
	No	85	59.11	<0.001*
2. I feel relaxed around people receiving palliative care	Yes	21	67.57	
	No	81	47.33	0.003*
3. I feel confident in managing symptoms in palliative care	Yes	21	58.12	
	No	83	51.08	0.299
4. I feel comfortable talking about dying to a patient receiving palliative care	Yes	21	58.36	
	No	82	50.37	0.251
5. I don’t mind working in palliative care despite its involvement in managing people with life-limiting illness	Yes	21	56.45	
	No	84	52.14	0.525
6. There is a difference between providing palliative care service and normal hospital care	Yes	21	49.40	
	No	85	54.51	0.457
7. I am not comfortable touching people with terminal illness	Yes	21	57.76	
	No	83	51.17	0.335
8. I don’t believe that pharmacists have any role to play as a member of palliative care team	Yes	21	53.12	
	No	85	53.59	0.940
9. I feel frustrated because I do not know how to help people receiving palliative care	Yes	21	58.10	
	No	83	51.08	0.303
10. It is not rewarding to work with people who are receiving palliative care	Yes	21	56.86	
	No	84	52.04	0.481
11. I am not familiar with pain symptoms necessary for palliative care	Yes	21	69.24	
	No	82	47.59	0.002*
12. Working with terminally ill patients is sad and depressing	Yes	21	58.76	
	No	83	50.92	0.267
13. Emotionally I don’t fit into palliative care	Yes	20	55.88	
	No	81	49.80	0.391

*N* Number, * Significant difference with Mann Whitney U (MWU) test. Higher mean rank for positive statements (1–6) indicate those who mostly agreed with the corresponding statement, while higher mean rank for negative statements (7–13) suggest those who least agreed with the corresponding statement, level of statistical significance *p* < 0.05

Hospital of practice by pharmacists significantly influenced their opinion on some attitude-related statements toward palliative care such as level of confidence in managing symptoms in palliative care, MR for FMC > OAUTHC > UCH [K-WX^2^ = 7.016, df = 2, two-tailed p value = 0.03] (see Table 7). Also, statement related to the extent of familiarity with pain symptoms in palliative care significantly differed among pharmacists from different practice sites, MR for FMC > OAUTHC > UCH (K-WX^2^ = 7.241, df = 2, two-tailed p value = 0.03). Years of experience in hospital practice, 1–10 versus > 10 years (Pearson X^2^ = 0.261, df = 1, two-sided *p* = 0.609), previous attendance in palliative care training or not (X^2^ = 0.108, df = 1, *p* = 0.74), gender (X^2^ = 0.563; df = 1, *p* = 0.453) and hospital of practice by pharmacists (X^2^ = 1.89, df = 2, *p* = 0.388) did not significantly influenced pharmacists’ general knowledge in palliative care.

**Table 7:** Association between pharmacists’ hospital of practice and attitude-related statements toward palliative care

Statement	Hospital of practice	N	Mean Rank	K-Wp-value
1.I enjoy working in palliative care	FMC	15	54.33	0.996
	OAUTHC	36	55.10	
	UCH	58	55.11	
2. I feel relaxed around people receiving palliative care	FMC	15	61.87	0.42
	OAUTHC	35	51.06	
	UCH	55	51.82	
3. I feel confident in managing symptoms in palliative care	FMC	15	71.93	0.03*
	OAUTHC	35	52.57	
	UCH	57	50.16	
4. I feel comfortable talking about dying to a patient receiving palliative care	FMC	15	53.17	0.37
	OAUTHC	34	58.50	
	UCH	56	49.62	
5. I don’t mind working in palliative care despite its involvement in managing people with life-limiting illness	FMC	15	69.20	0.02*
	OAUTHC	35	58.83	
	UCH	58	48.09	
6. There is a difference between providing palliative care service and normal hospital care	FMC	15	46.57	0.49
	OAUTHC	35	57.04	
	UCH	58	55.02	
7. I am not comfortable touching people with terminal illness	FMC	15	64.73	0.18
	OAUTHC	36	48.47	
	UCH	56	54.68	
8. I don’t believe that pharmacists have any role to play as a member of palliative care team	FMC	15	61.57	0.43
	OAUTHC	35	50.94	
	UCH	58	54.82	
9. I feel frustrated because I do not know how to help people receiving palliative care	FMC	15	63.43	0.35
	OAUTHC	35	54.31	
	UCH	57	51.32	
10. It is not rewarding to work with people who are receiving palliative care	FMC	15	54.37	0.88
	OAUTHC	35	56.47	
	UCH	58	51.32	
11. I am not familiar with pain symptoms necessary for palliative care	FMC	14	66.43	0.03*
	OAUTHC	35	58.31	
	UCH	58	46.32	
12. Working with terminally ill patients is sad and depressing	FMC	14	65.29	0.27
	OAUTHC	35	58.31	
	UCH	58	46.32	
13. Emotionally I don’t fit into palliative care	FMC	15	59.57	0.31
	OAUTHC	33	54.73	
	UCH	55	48.27	

*N* Number, * Significant difference with Kruskal-Wallis (K-W) test. Higher mean rank for positive statements (1–6) indicate those who mostly agreed with the corresponding statement, while higher mean rank for negative statements (7–13) suggest those who least agreed with the corresponding statement. Level of statistical significance *p* < 0.05. *FMC* Federal Medical Centre, *OAUTHC* Obafemi Awolowo University Teaching Hospitals Complex, *UCH* University College Hospital

## Discussion

In this study, we comprehensively evaluated the knowledge, attitude and involvement of hospital pharmacists in selected tertiary care institutions in Nigeria in palliative care, while factors that may hinder their participation in palliative care were also explored. Notably, a response rate of 100% was recorded which may possibly be an indication that the research area for our study might be of interest to the participants. However, 78.9% of the pharmacists had inadequate general knowledge of palliative care, with almost two-thirds who had a misconception that medication therapy is the cornerstone of all symptom control in palliative care. More than 90% identified the goals of palliative care to be consistent with the philosophy of pharmaceutical care, while all recognized the fact that pharmacists’ involvement in palliative care may decrease the need for medical emergencies. O’Connor et al [30] in the Australian nationwide survey reported that community pharmacists’ participant in their study had good knowledge of some aspects of palliative care but misconception about others. Some studies from developed countries have also identified low level of knowledge of palliative care among pharmacists [14–16, 38]. However, perusing the response of our study participants to the knowledge questions gave highlights on the areas of strength and common misconception about palliative care, thereby indicating the possible aspects to focus for educational programme and future intervention to address the knowledge gaps among the pharmacists.

It is worthy of note to mention that less than one-fifth of the pharmacists had previous training in palliative care, with less than 1% who reported to have worked as a member of palliative care team, but more than three-quarters had encountered patients requiring palliative care service. This perhaps underscore the need to further encourage pharmacists generally and hospital pharmacists in particular to engage in palliative care related training, in order to ensure effective contribution in palliative care. Previous studies have identified the importance of palliative care training in improving the knowledge of pharmacists, as well as facilitating their involvement in providing services for palliative care patients [21, 30]. Pharmacists’ participation as a member of palliative care team can potentially improve patient medication management knowledge, as well as reduce the risk of non-adherence [14, 15, 38]. Studies especially in developed countries have identified and reported pharmacists’ positive contributions in palliative care [15, 18–21, 39].

Overall, approximately one-eighth demonstrate positive attitude towards palliative care, with slightly more than half who felt confident in managing symptoms in patients receiving palliative care, while 41.3% enjoy working in palliative care. Research indicates that many healthcare professionals especially in developing countries are poorly prepared for the complexities of palliative care [21, 28, 29, 33], with the key factors influencing involvement mentioned to include confidence issues, inadequate knowledge and skills as well as attitude, belief and experience [16, 19, 30, 33]. Thus, any palliative care educational programme or training to be designed for pharmacists should largely focus on building knowledge as well as fostering positive attitude and belief about palliative care [30], as this may help to increase involvement in palliative care services [40, 41]. Belief, support and knowledge have been reported as predictors of pharmacists’ overall positive attitude towards palliative care [30]. One measure to ensure increase in pharmacists’ knowledge and attitude about palliative care, especially in resource-poor countries may partly involve incorporation of palliative care concept into the curriculum of undergraduate and postgraduate pharmacy education, while the mandatory continuous professional development programme for practicing pharmacists should also include aspects detailing the fundamental principles of palliative care.

In this study, around a third of pharmacists reported that they are comfortable working in palliative care, with only about one-eighth who felt at ease talking about dying to patients receiving palliative care. The multifaceted nature of palliative care require professionals working with patients having life-limiting illness to possess the ability to address psychosocial needs [42, 43], through effective communication skills [21]. Healthcare providers including pharmacists therefore need to develop excellence in communication skills that will assist in clarifying the psychological and social needs of the patients so as to consistently ensure better therapeutic outcomes.

Nearly half of the pharmacists agreed with the statement that indicates that it is sad and depressing working with terminally ill patients. Even though it may be expected that managing patients with life-limiting illness especially at the end of life can be emotionally draining [2, 5, 6], palliative care providers should not allow his/her emotion to affect the therapeutic relationship with the patients, thus the need to develop appropriate communication skills and approach to handle the psychosocial aspect of care. Palliative care is an approach to care that emphasize the relief of patients from all forms of suffering and pain including physical, psychological, social, as well as spiritual discomfort [2, 6–8]. In addition to possessing effective communication skills by the pharmacists, clinical experience especially in pain management and symptom control is equally important. Joranson and Gilson [44] report on the influence of pharmacists’ knowledge and attitude to opioid pain medications and concluded that incorrect knowledge and inappropriate attitude could lead to errors in the service provided for palliative care patients. Interestingly, in our study, pharmacists who had training in palliative care were largely found to be familiar with pain symptoms in palliative care compared to those without training. Studies have reported improved confidence and positive effects especially in pain and symptom management among healthcare professionals after attendance of post-qualification courses and educational intervention in palliative care [15, 16, 45–47]. Therefore, exposing the future and practicing pharmacists to the core principles of palliative care may perhaps instill the confidence and other needed skills to effectively serve as a competent member of palliative care team. However, to ensure effective palliative care service that will improve the quality of care for patients, palliative care educational programme and training should encompass comprehensive pain management techniques including assessment, dose conversion, and counselling especially to allay fear of addiction associated with opioids, as well as development of treatment plans in accordance with accepted standards [13, 14].

More than 90% each of the pharmacists cited counselling on therapy adherence as well as ensuring complete labelling and direction for medication usage as the most frequently engaged activities, while attending clinical meetings and providing educational sessions for other members of healthcare team were mentioned as occasionally performed duties. Patient home visit was topmost of the duties not done at all. Pharmacists’ occasional engagement in some of the core practice roles that may propel team-based care possibly suggests the need to also focus palliative care educational programme and training on aspects that will encourage and facilitate collaboration with other healthcare team. Delivery of excellent palliative care requires collaborative input of physicians, pharmacists, nurses and psychosocial careers in a holistic framework, thereby fostering increased confidence with improved quality of care to patients [48, 49]. The low level of pharmacists’ involvement in patient home visit as reflected in our study may also be an issue that need to be improved upon if pharmacists intend to be actively involved in the provision of palliative care service. This is largely because the care for people with life-limiting illness especially in the final year of life is increasingly being moved from hospital to the home [50]. Home-based care has been reported as the most common setting where approximately 70 to 80% of terminally ill patients prefer to receive care [51].

Topmost of the perceived factors limiting involvement of pharmacists in palliative care was reported as pharmacists’ unawareness of their need in palliative care, while lack of access to patients’ medication profile, as well as inadequate knowledge of palliative care were also mentioned as barriers. Limited access to patients’ medical records has been generally reported as one of the major factors hindering pharmacists’ proactive engagement in patient-centred care such as palliative care [18, 52]. However, confidence issues [18] as well as inadequate knowledge and skills necessary for delivering effective palliative care service [16, 30, 33] remains a paramount gap that need to be addressed among pharmacists generally, and hospital pharmacists in particular on account of their strategic role in healthcare delivery. Contrary to what may be obtained in most developed countries [18–20, 53], in Nigeria, patients who require palliative care mostly attend tertiary care hospitals [24], thereby encounter hospital pharmacists more often than those in other practice areas. Therefore, efforts should be geared towards ensuring increase in knowledge and skills of hospital pharmacists in medical ethics, pain management, as well as symptom control with excellence in communication skills [43, 54]. This can be partly achieved through a focused and targeted educational and training programme towards the areas of deficiencies and needs among the pharmacists.

In this study, opinion on some attitude-related statements significantly differ among pharmacists from the three hospitals, especially in relation to the level of confidence in managing symptoms in palliative care, as well as flair for involvement in palliative care services. In Nigeria, palliative care is an emerging specialty, thus, healthcare professionals in each hospital may possibly be at different stages of palliative care involvement and implementation. The American Society of Health System Pharmacists has stated that the extent of service rendered by pharmacists may differ based on practice experience as well as the level of palliative care services in the respective setting [16].

Though, this study provides useful information about hospital pharmacists’ knowledge, attitude and involvement in palliative care, its limitation may include the small sample size, though representative of the pharmacists’ population in the studied hospitals. Also, only content validity and pretest of the study instrument were done rather than subjecting the questionnaire to all the validation checks typically require for a health-related patient reported outcome [55], thus, there may be possibility of bias with some questions or statements. Nevertheless, this study focused largely on hospital pharmacists’ knowledge, attitude and involvement in palliative care, thus, the rigorous validation checks may not be strictly essential [55]. Moreover, the item-statements in the questionnaire for our study covers a wide-range of aspects in palliative care which may partly allow for a comprehensive exploration of participants on the subject area, hence, a useful strength for our study. Another limitation may be linked to the fact that, our study was carried out in tertiary hospitals with established palliative care services, thus the need for caution in generalising the findings to the entire population of hospital pharmacists in the region.

## Conclusion

It can be concluded from this study that hospital pharmacists in selected tertiary care institutions demonstrate inadequate general knowledge, as well as negative attitude towards palliative care. Also, their involvement in core palliative care service is generally low, with pharmacists’ unawareness of their need in palliative care constituting a major barrier. This perhaps suggest a need for inclusion of palliative care concept into the pharmacy education curriculum, while mandatory continuous professional development programme for practicing pharmacists should also incorporate aspects detailing fundamental principles of palliative care, in order to bridge the knowledge and practice gaps.

## Supplementary information

**Additional file 1.** Questionnaire for the study.

## Data Availability

The datasets used and/or analysed during the current study are available from the corresponding author on reasonable request.

## Abbreviations

EC: Ethics committee
FMC: Federal Medical Centre
K-W: Kruskal Wallis; MR: Mean rank
MWU: Mann-Whitney-U
OAUTHC: Obafemi Awolowo University Teaching Hospitals Complex
PC: Palliative care
SPSS: Statistical package for social sciences
UCH: University College Hospital
UI: University of Ibadan
