# Supplementary material for: Pharmacists’ knowledge, attitude and involvement in palliative care in selected tertiary hospitals in southwestern Nigeria
Source: BMC Palliat Care. 2019 Nov 29;18:107. doi: 10.1186/s12904-019-0492-8 (PMC6884848; doi:10.1186/s12904-019-0492-8)
Supplement: Supplementary file 1 — Additional file 1. Questionnaire for the study. [file 12904_2019_492_MOESM1_ESM.doc]

**UNIVERSITY OF IBADAN**

**FACULTY OF PHARMACY**

**DEPARTMENT OF CLINICAL PHARMACY**

**QUESTIONNAIRE FOR THE PHARMACISTS**

Dear respondent,

This questionnaire is designed to elicit information on knowledge, attitude and involvement in palliative care among hospital pharmacists. This study is carried out purely for academic purposes and the information given will be treated with utmost confidentiality and anonymity, while the responses will be used for only the purpose of this study. It is therefore requested that you spare only few minutes of your time in answering the questionnaire.

Thank you for your support and cooperation

**SECTION A**

***Tick or write appropriately in the space provided as it applies to you***

**Section A: Socio-demographics and practice information**

1. Age: 20-30 ( ) 31-40 ( ) 41-50 ( ) 51-60 ( ) >60

2 .Gender: Female [ ] Male [ ]

3. Highest level of pharmacy qualification completed? Associate's degree ( ) Bachelor’s degree ( ) Master’s Degree ( ) Doctoral degree ( ) Others, kindly specify( )

4. How many years have you been in the hospital practice? ……………………………..

5. Level or cadre: Pharmacist I ( ) Senior Pharmacist ( ) Principal Pharmacist ( ) Chief Pharmacist ( ) Assistant Director ( ) Others, kindly specify……….

6. Sections you have worked in the past 2 years (a)……………………………………………

(b)-------------------------- (c)………………………………..(d)…………………

( e)……………………… (f)……………………………….

7. Have you attended a training related to palliative care? Yes ( ) No ( )

If yes, when:………………………………… where ? ………………………………

8. Have you ever attended a conference or in-service program on palliative care or related to palliative care? Yes ( ) No ( )

9. Have you encountered patients requiring palliative care in your practice? Yes ( ) No ( )

**Section B: Knowledge test questions in palliative care and its key principles**

The statement below relates to palliative care and its key principles. Kindly answer the following questions to the best of your ability by ticking the appropriate response (True or false) that corresponds with your answer.

|  | Statement | True | False |
| --- | --- | --- | --- |
|  | Palliative care involves provision of care only to patients who have no curative treatments available |  |  |
|  | Non-medical practitioners are active participants in palliative care |  |  |
|  | Palliative care is to be provided by doctors and nurses alone |  |  |
|  | Palliative care is required only for patients who are near death |  |  |
|  | Palliative care only involves pain management |  |  |
|  | Palliative care involves providing patients with relief from their symptoms |  |  |
|  | Regular opioids intake should not be combined with non-steroidal anti-inflammatory drugs for palliative care patients |  |  |
|  | Long term use of opioids for palliative care patients does not often induce addiction |  |  |
|  | Palliative care should not be provided alongside anti-retroviral treatment |  |  |
|  | One of the goals of pain management in palliative care is to get good night sleep |  |  |
|  | Benzodiazepines should be effective for controlling delirium in palliative care patients |  |  |
|  | Palliative care does not involve maintaining patient’s medication profile overtime |  |  |
|  | Palliative care should not be provided in conjunction with curative care at the time of diagnosis of a potential life-limiting illness |  |  |
|  | The goals of palliative care and pharmaceutical care are consistent |  |  |
|  | Medication therapy is the cornerstone of all symptom control in palliative care |  |  |
|  | Involvement in palliative care activities by pharmacists may decrease the need for medical emergencies. |  |  |
|  | Pharmacist in palliative care should be less concerned about monitoring non-prescription medication use for safety and effectiveness |  |  |
|  | Pharmacists in palliative care communicate with pharmaceutical manufacturers to determine the availability of nonstandard dosage forms. |  |  |

**Tick appropriately to the best of your understanding:**

**Various disease(s) requiring palliative care**

19. The under listed conditions will require palliative care service: Cardiovascular diseases ( ) HIV/AIDS ( ) Renal diseases ( ) Peptic Ulcer Diseases ( ) Asthma ( ) End stage pulmonary diseases ( ) Parkinson disease ( ) Dementia ( )

**Professionals that can constitutes palliative care team composition, you can tick more than one option**

20. Doctors ( ) Pharmacist ( ) Nurses ( ) Psychologist ( ) Chaplain ( ) Social workers ( ) All of the above ( )

**Section C: Attitude towards palliative care**

The statements below relate to palliative care, kindly tick the column that best corresponds with your opinion or attributes following the response options: Strongly Agree, Agree, Undecided Disagree and Strongly Disagree.

|  | **Item-statement** | **Strongly**  **disagree** | **Disagree** | **Undecided** | **Agree** | **Strongly agree** |
| --- | --- | --- | --- | --- | --- | --- |
|  | I enjoy working in palliative care |  |  |  |  |  |
|  | I feel relaxed around people receiving palliative care. |  |  |  |  |  |
|  | I feel confident in managing symptoms in palliative care |  |  |  |  |  |
|  | I feel comfortable talking about dying to a patient receiving palliative care |  |  |  |  |  |
|  | I don’t mind working in palliative care despite its involvement in managing people with life-limiting illness |  |  |  |  |  |
|  | There is a difference between providing palliative care service and normal hospice care |  |  |  |  |  |
|  | I am not comfortable touching people who have terminal illness |  |  |  |  |  |
|  | I don’t believe that pharmacists have any role to play as a member of palliative care team |  |  |  |  |  |
|  | I feel frustrated because I do not know how to help people receiving palliative care. |  |  |  |  |  |
|  | It is not rewarding to work with people who are receiving palliative care |  |  |  |  |  |
|  | I am not familiar with the experiences of various pain symptoms necessary for palliative care |  |  |  |  |  |
|  | Working with terminally ill patients is sad and depressing. |  |  |  |  |  |
|  | Emotionally I don’t fit into palliative care |  |  |  |  |  |

**Section D**: **Clarify the extent of** **pharmacists’ involvement in general and palliative care services**

The statement below intends to clarify the extent of your involvement in patient care activities in your practice setting. Kindly response with sincerity by ticking the correct option as applicable to you using the appropriate response options: Not at all, Rarely, Occasionally, Frequently

|  | Question-items | Not at all | Rarely | Occasionally | Frequently |
| --- | --- | --- | --- | --- | --- |
|  | How often do you explain misconceptions about addictive medication? |  |  |  |  |
|  | How often do you visit patients’ homes to communicate directly with patients and their caregivers and to make necessary assessments? |  |  |  |  |
|  | How often do you monitor patient’s medication profile for safety and effectiveness |  |  |  |  |
|  | How often do you provide patients with essential medications that ensures continuous symptom control |  |  |  |  |
|  | How often do you attend clinical meetings to advise other team members about medication therapy |  |  |  |  |
|  | How often do you advise clinical teams on dosage forms and adjustment, routes of administration, costs, and availability of various drug products? |  |  |  |  |
|  | How often do you give educational sessions? |  |  |  |  |
|  | How often do you advise members of the clinical teams about the potential for toxicity and interactions with dietary supplements and alternative therapies? |  |  |  |  |
|  | How often do you ensure that all medication labeling is complete and understandable by patients and their caregivers? |  |  |  |  |
|  | How often do you communicate with patients about the importance of adhering to the prescribed drug regimen? |  |  |  |  |
|  | How often do you monitor all prescription and nonprescription medication use? |  |  |  |  |
|  | How often do you counsel patients about potential toxicity of alternative and complementary therapies? |  |  |  |  |
|  | How often do you or the hospital engage in extemporaneous preparation of non-conventional dosage forms for ease of administration to patients |  |  |  |  |
|  | How often do you prepare flavouring medications to promote compliance |  |  |  |  |
|  | How often do you address issues on cost of patients’ medications? |  |  |  |  |
|  | How often do you ensure that drug disposal is in compliance with federal and state drug control and environmental protection laws and regulations? |  |  |  |  |

**Section E: Barriers/factors limiting pharmacist’s involvement in palliative care**

In your own opinion what are the likely barriers to pharmacists’ involvement in palliative
care? Tick the appropriate column as it applies to you.

|  | Items | Yes | No | Don’t Know |
| --- | --- | --- | --- | --- |
|  | Confusion of role in palliative setting |  |  |  |
|  | Lack of awareness of the need for pharmacists in palliative care |  |  |  |
|  | Lack of pharmacists’ interest to work in palliative care |  |  |  |
|  | Fear of being around people with terminal illness |  |  |  |
|  | Lack of reimbursement |  |  |  |
|  | Lack of access to patients medication profile |  |  |  |
|  | Belief that there could be a spiritual backlash from engaging in palliative care |  |  |  |
|  | Inadequate knowledge of concept of palliative care |  |  |  |
|  | Inadequate knowledge of palliative care among pharmacists |  |  |  |

**Others not listed above: __________________________________________________________________________________________________________________________________________________________________________________________________________________________________________**
